# Ethyl Chlorid—A General Anesthetic for Minor Operations

**Published:** 1907-06

**Authors:** Clayton F. B. Stowell

**Affiliations:** Flint, Mich.


### ETHYL CHLORID—A GENERAL ANESTHETIC FOR MINOR OPERATIONS

Clayton F. B. Stowell, D. D. S., Flint, Mich.

The amount of ethyl chlorid used in this country by dentists, rhinologists and by general surgeons for minor operations is steadily increasing. The fact that its use is very generally unaccompanied by fatal or even mildly undesirable conditions of the patient is constantly inducing more anesthetists to adopt this drug.

"Be not the first by whom the new is tried
Nor yet the last the old to lay aside."

Ethyl chlorid has had many trials and has proved eminently satisfactory. The only thing I wonder at in connection with this anesthetic is that in the face of the fact that its effects are so salutary, it has been so studiously overlooked in the observations by our research men. I dislike to say it, even our college faculties have exploited and boomed proprietary preparations far less commendable, and never have they given ethyl chlorid an hour's consideration in the class-room.

Ethly chlorid chemically is a halogen derivative, it being derived from ethly alcohol by substituting for the hyden-oxyl group an atom of chlorin; it is a clear colorless liquid and smells something like chloroform; it is very combustible, boils at 12.5 C. and evaporates at room temperature, therefore a carefully sealed tube should contain it or you will find your container empty too often.

Ethyl chlorid has no exciting effect upon the respiratory mucous membrane; very little irritation is ever noticed. This is a point of great importance and one which makes it possible to administer it in asthmatic cases and where nitrous oxid would be contradicted.

Somnoform has only one advantage over ethyl chlorid. Narcosis is produced in less time, owing to the ethyl bromid it contains, which to offset this advantage be it said is a very unstable compound and apt to contain free bromine. Ethyl chlorid anesthesia has been continued for fifty-seven minutes by Manwaring. The period of induction varies from forty-five seconds to two minutes. Statements in regard to this are misleading and of very little moment.

Alcoholics and neurotics take ethyl chlorid badly.

In induction the eye is a good indicator of the stages of anesthesia, or the uplifted hand may by dropping indicate that anesthesia is occuring; the operator should watch that reflex with which he is most familiar.

There is no cynosis with ethyl chlorid anesthesia, so that friends of the patient who may by chance be at hand will not become alarmed. The apparatus is very convenient and not at all appalling to the nervous patient. The drug is not unpleasant, rarely does it cause nausea. The headache following the administration of somnoform is not present, the bromide of chlorin in this anesthesia being not only useless but harmful.

Ethyl chlorid is recommended to the dentist for carefully selected cases; it can be administered and with safety to many patients where other anesthetics are plainly contraindicted; such as high degrees of circulatory interruption, (2) fatty degeneration of the heart, (3) persons enfeebled by a great loss of blood, (4) disease of the respiratory organs, (5) persons suffering from nervous shock.

Cumiston asserts that ethyl chlorid may be given to patients of any age, particularly the extremes when the organism does not as yet present, or, on the other hand, has lost that vitality which is requiste to support the shock of our other anesthetics.

The only contraindications for the use of ethyl chlorid may be summed up thus: Inflammation or narrowing of the larynx and a swelling or edema about the larynx. I would use it in any case I thought safe for nitrous oxid. —Northwestern Dental Journal.

